# Unravelling the secret of seed-based gels in water: the nanoscale 3D network formation

**DOI:** 10.1038/s41598-018-25691-3

**Published:** 2018-05-09

**Authors:** Malick Samateh, Neethu Pottackal, Setareh Manafirasi, Adiyala Vidyasagar, Charles Maldarelli, George John

**Affiliations:** 1 Department of Chemistry and Biochemistry & Center for Discovery and Innovation (CDI), The City College of New York, New York, NY 10031 USA; 2 Ph.D. Program in Chemistry, The Graduate Center of the City University of New York, New York, NY 10016 USA; 3 Department of Chemical Engineering, The City College of New York, New York, NY 10031 USA

## Abstract

Chia (*Salvia* hispanica) and basil (*Ocimum* basilicum) seeds have the intrinsic ability to form a hydrogel concomitant with moisture-retention, slow releasing capability and proposed health benefits such as curbing diabetes and obesity by delaying digestion process. However, the underlying mode of gelation at nanoscopic level is not clearly explained or explored. The present study elucidates and corroborates the hypothesis that the gelling behavior of such seeds is due to their nanoscale 3D-network formation. The preliminary study revealed the influence of several conditions like polarity, pH and hydrophilicity/hydrophobicity on fiber extrusion from the seeds which leads to gelation. Optical microscopic analysis clearly demonstrated bundles of fibers emanating from the seed coat while in contact with water, and live growth of fibers to form 3D network. Scanning electron microscope (SEM) and transmission electron microscope (TEM) studies confirmed 3D network formation with fiber diameters ranging from 20 to 50 nm.

## Introduction

Gels, from the body of a jellyfish and bacterial cell wall to the pith of the aloe vera plant, are ubiquitous in nature and have expedient applications in a plethora of areas such as in food, cosmetics and drug-delivery. Probing the process of gelation is a passion and a key topic of research in our group^1–11^. The gelation studies entail designing low-molecular-weight gelators (LMWGs) that have the propensity to hierarchically self-assemble via non-covalent interactions into a 3D self-assembled fibrillar network (SAFiN). The nanoscale SAFiN entraps the solvent to form a gel known as a molecular gel (MG). The entrapment and immobilization of a solvent pool (water, organic solvent or oils) into a gel by a nanoscale 3D network of synthetic gelators has been well documented^12–17^.

**Figure 1:**
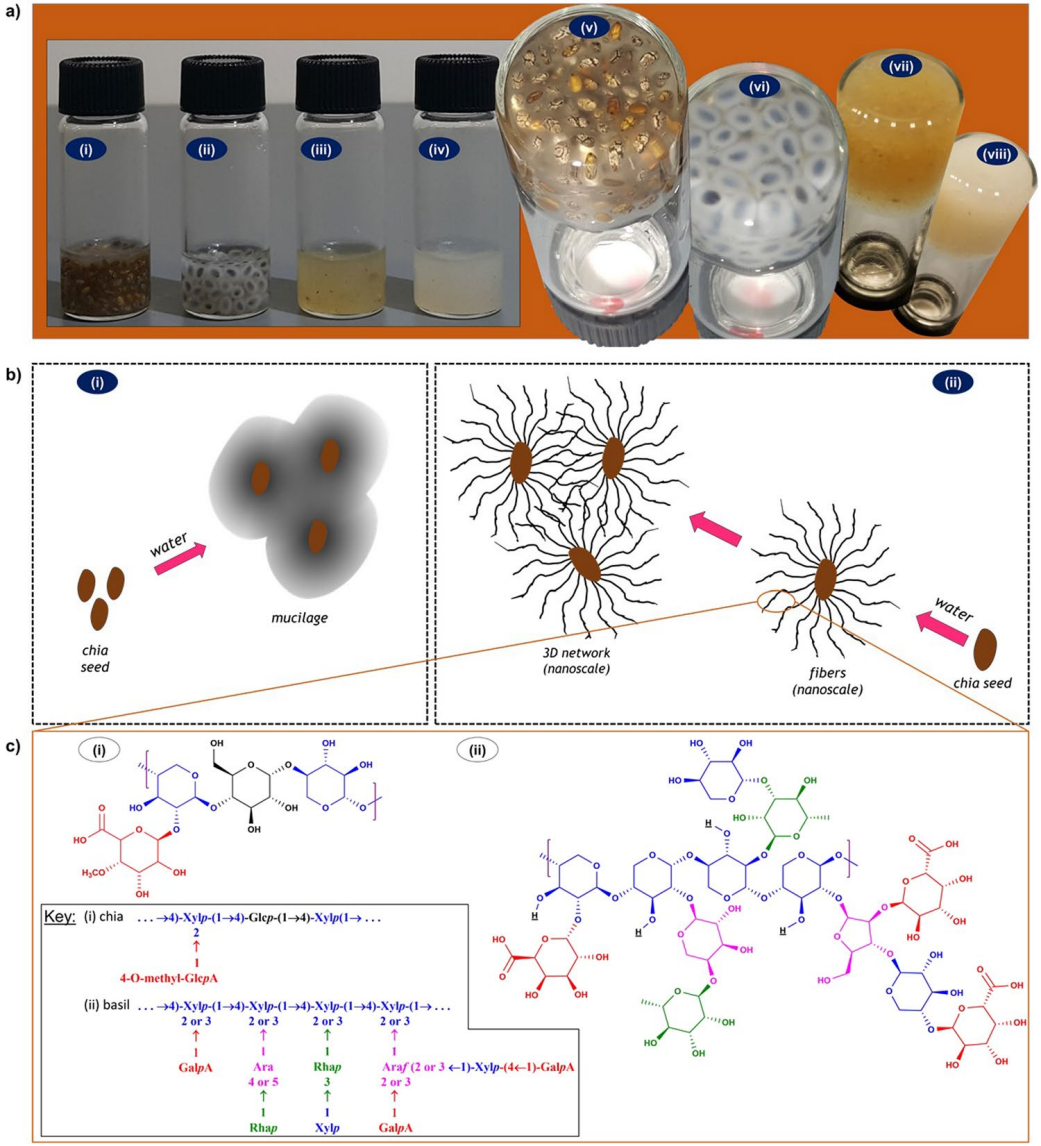
(**a**) Chia and basil gels; seed gels: (i) chia, (ii) basil; mucilage gels: (iii) chia, (iv) basil; upside down: (v, vii) chia, (vi, viii) basil. (**b**) Illustration of gelation based on the current notion and our hypothesis; (i) Current notion: gel formation is merely due to the seed’s mucilage swelling in or coagulating water; (ii) Hypothesis: mucilage of chia contains nanoscale fibers that extend in water to form 3D network, entrapping and “congealing” water to form a gel. (**c**) chemical structures of reported compositions^20,21^: (i) chia repeating oligomer; (ii) basil repeating oligomer.

**Figure 2:**
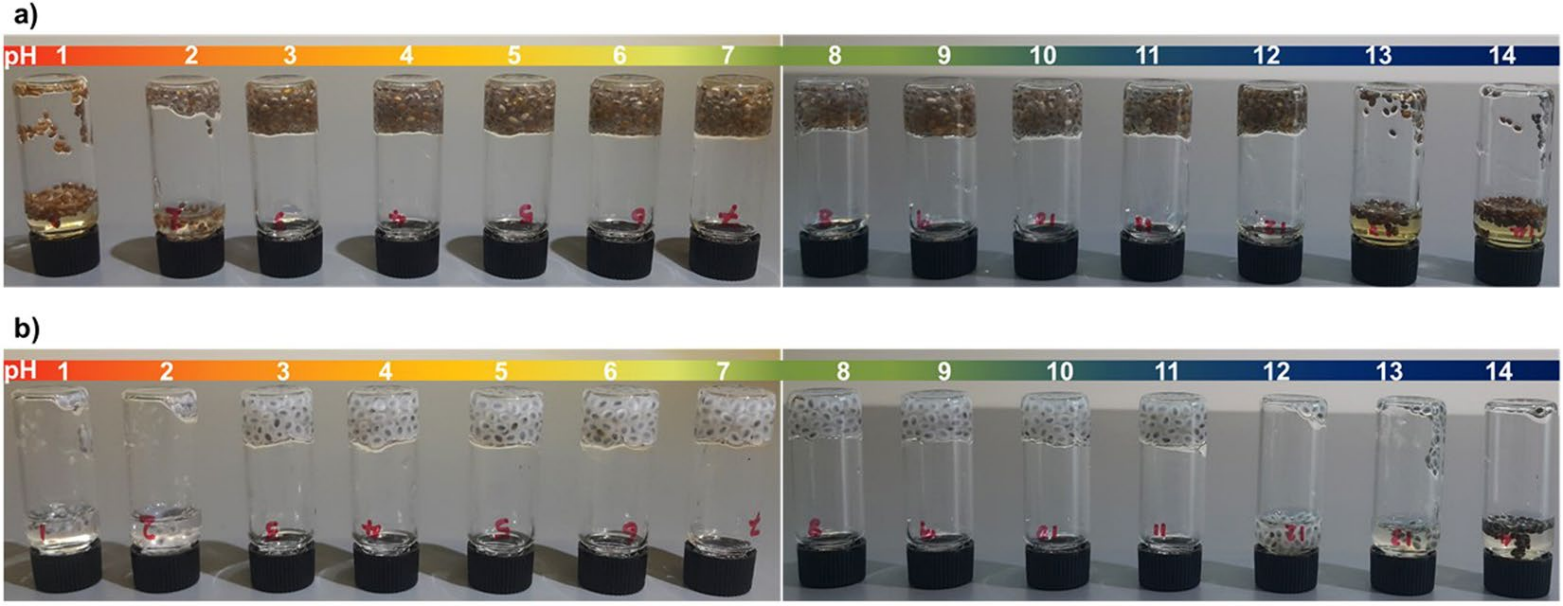
Photographs showing gelation of chia and basil gels in different pH solutions – pH 1 to 14: (**a**) 15% w/v chia seed in respective pH solutions; (**b**) 6% w/v basil seed in respective pH solutions.

Interestingly, chia (*Salvia* hispanica) and basil (*Ocimum* basilicum) are inherently predisposed to undergo the process of gelation in water (Figs 1a and 2) without the intervention of chemical design and synthesis, prompting our scientific curiosity to probe their mode of gelation. Both chia and basil seeds are annual herbaceous crops of the Lamiaceae family, known to be the ancient food of the future. Chia is used as a nutritional supplement and to produce cereals, bars and cookies, while basil is commonly used in a variety of ways including as food, culinary herb and traditional medicine^18,19^. Chia and basil are covered (coated) with polysaccharides: a tetramer of glucose, xylose and glucuronic acid in a 1:2:1 ratio, for chia^20^, and primarily xylose, arabinose, rhamnose, and galacturonic acid in a 15:9:7:12 ratio, for basil^19,21^ (Fig. 1c). The ability of these natural materials like chia and basil to produce gel/mucilaginous substance has vital ecological functions such as self-propagation, stability during flooding and survival in harsh environmental conditions^22–24^. The exceptional water retaining properties have imparted to the seeds desirable qualities that have key applications such as food thickening and emulsion preparations^25,26^. The mucilaginous jelly substance has been reported to slow down digestion and prolong the release of glucose or food into the bloodstream^25–27^. This property may attribute to multiple health benefits; alleviating obesity, over-eating, and other similar diet-related health problems including type II diabetes and its complications^25–28^.

In this study, we intended to affirm the resemblance between the nature of the gel network formed from these seeds with that formed from synthetic gelators. We questioned the adequateness of the current notion that seeds like chia and basil form a gel in water merely by the mucilage coagulating or swelling in water **(**Fig. 1b(i)); we deemed the explanation to fall short of shedding enough light on the gelation mechanism at nanoscale level. We henceforth hypothesized that the mucilage of such seed contains fibrous materials/strands that extend out in the presence of water and interconnect with those of other nearby seeds to form a nanoscale 3D network (Fig. 1b(ii)); the network “congeals” water to form a gel just as seen with SAFiNs in the case of LMWGs. The goal was to unravel the fundamental process and demystify the hydrogelation of the seeds at microscopic level. Although the composition and gelation of such seeds have been reported (Fig. 1c)^20,21^, to the best of our knowledge, an investigation to reveal the nanoscale fiber and the assembled 3D architectures associated with the gelation of such seeds remains unexplored. The main tool used to verify and confirm our working hypothesis involved using various microscopic techniques; however, we began with preliminary gelation studies to deduce and understand how various parameters like solvent polarity and pH influenced the network formed by the mucilage. Optical, scanning electron and transmission electron microscopes were subsequently used for imaging. Optical microscopy was used to zoom in and elucidate how the fibers grew around the seed in water. Scanning and transmission electron microscopies were then used to explore the nature of the fibers, particularly size and configuration, in the network at nanoscale. This knowledge will inevitably be important in materials design and further health benefits associated with consumption of such seeds^29,30^. Exploring intricate natural processes and nature’s responsive mechanisms is vital in the process of bio-inspired innovations and future bio-interface materials discovery. Therefore, an understanding of the gelation and gelation mechanism of natural seeds has foreseeable potential for the development of improved, functional food materials that have similar benefits as seen with chia and basil.

## Results and Discussion

### Effects of different parameters on gelation

The preliminary study enabled discerning the nature of the material covering the surfaces of seeds such as that of chia and basil via probing how the seeds behaved as each of several gelation parameters was varied. Figure 1a shows typical hydrogels of the seeds as well as their mucilages.

The results of how various parameters influence the gelation of chia and basil seeds are tabulated in Table 1. The ratio dependent experiment has shown that both chia and basil seeds were quite compatible with water and the feasibility of gel formation increased with increasing seed-to-water ratio. Chia formed hydrogel at seed-to-water ratio of 15% or higher while basil formed hydrogel at seed-to-water ratio of 6% or higher; below the respective seed-to-water ratios, the seeds merely formed partial gels and did not formed self-standing gels. Hence, 15% w/v and 6% w/v are the minimum gelation ratios (MGRs) of chia and basil, respectively, as shown in Table 1a. This observed MGR difference between the seeds suggests basil to be a more effective gelator and implies that the type of seed used is as equally important factor as the amount of seed in hydrogelation of these seeds. This conspicuously enhanced gelation capability of basil seed is attributed to the repeating units in basil having more branches compared to chia as seen in Fig. 1c. Chia is a linear polymer with only one sugar unit as the side chain on the sugar backbone, whereas in basil every sugar unit in the backbone is attached to a side chain that is monomeric, dimeric or tetrameric in nature. The more complex branching in basil leads to an efficient 3D network and better gelation capacity.

**Table 1:** The effects of various gelation parameters on gelation of chia and basil seeds.

Substance	(a) Seed-to-water ratio	(b) Polarity: %EtOH in H_2_O				(c) Mode of adding 50:50 H_2_O/EtOH			(d) pH					(e) Hydrophobic solvent	
	MGR (% w/v)	0	10, 20	30, 40	50–100*	Water 1^st^	together	EtOH 1st	1	2	3–11	12	13, 14	hexane	canola
chia seed	15	G	N	N	N	PG	N	N	N	PG	G	G	PG	N	N
chia mucilage	1.5	—	—	—	—	—	—	—	—	—	—	—	—	—	—
basil seed	6.0	G	G	PG	N	PG	N	N	N	PG	G	PG	PG	N	N
basil mucilage	1.2	—	—	—	—	—	—	—	—	—	—	—	—	—	—

G = gel; PG = partial gel; N = no gel; MGR = minimum gelation ratio; EtOH = ethanol; *50, 60, 70, 80, 90, 100%.

The results presented in Table 1a also indicate that small amount is needed when mucilage is used instead of the whole seed; that is, MGR values of only 1.5 and 1.2% w/v were needed when the mucilages were used as opposed to 15 and 6% when whole seeds were used for chia and basil, respectively. This is consistent with the fact that the mucilage is only a small fraction of the whole seed.

The effect of polarity on seed gelation was studied by progressively using increasing amounts of ethanol in water (Table 1b). Using deionized water resulted in gelation in the cases of both seeds. For chia, 10 to 100% ethanol in water resulted in no gel formation. For basil, 10 and 20% ethanol in water resulted in gel formation with a few droplets of water dripping upon inversion of the vial, 30 and 40% ethanol in the mixture resulted in partial gel, and 50 to 100% ethanol in the mixture resulted in no gel formation. Similar results were obtained using methanol instead of ethanol. These results suggest a polar nature to the gel-forming agent on the surface of the seeds, which are more compatible with more polar solvents.

Our investigation also showed the order the water and ethanol were added in their mixture influenced whether the seeds partially gelled or not (Table 1c). Adding water first led to partial gelling of the seeds while adding ethanol first did not lead to gelation at all. This can be explained by the fact that adding water first induced the process of gelation, which was arrested as soon as ethanol was added. Adding water and ethanol simultaneously did not give any observable gelation due to the solvent mixture’s lower polarity as mentioned earlier. As observed with effect of polarity study, methanol/ethanol solvents yielded analogous outcomes.

As shown in Table 1d and Fig. 2, chia seeds formed gel at pH 3 to 12 and a partial gel at pH 2, 13 and 14; and did not gel at pH 1. Basil seeds formed gel at 3 to 11, partially gelled at pH 2, 12, 13 and 14, and did not gel at pH 1. The polarity and pH studies seem to suggest that basil fared better in less polar solvent systems than chia did; it gelled or partially gelled in up to 40% ethanol solvent system whereas chia had only negligible gelation ability in 10 and 20% ethanol solutions. This implies the composition of the gelling agent (the mucilage) of basil to be slightly less polar than that of chia.

Interestingly, our investigations have revealed that, once the seeds were allowed to first fully or partially form gel in water, the addition of ethanol or highly acidic or basic solution did not have much effect on the already-formed gel or partial gel despite lack of compatibility with ethanol or extreme pH solution. The expectation was that, once added to the mixture of seed and water, the ethanol or extreme pH solution would diffuse into the existing water, changing the native polarity and hence leading to the shrinkage of the mucilage or the weakening of the formed gel. Not observing the anticipated result could be logically attributed to the fact that the extended fiber in the mucilage clung tightly to a layer of water surrounding the fiber, preventing that layer from freely mixing with the added ethanol and hence retaining the original polarity at the immediate vicinity of the fiber; in other words, the layer of water at the proximity of the fibers does not partition freely into the added ethanol. Moreover, adding highly acidic (pH 1) solution to a formed or partially formed gel in water showed similar result; the acidic solution did not significantly disrupt the already gelled or partially gelled seeds in water.

Table 1e indicates that hydrophobic solvents like pure hexane and canola oil did not lead to gelation. As expected, this is consistent with the reported polar composition of the mucilage.

Extraction of the mucilage—via soaking, freezing, freeze-drying, and gently rubbing-off—revealed that about 10% of chia seed or 21% of basil seed were made up of the mucilage. This agreed with the fact that only a minimum (or MGR value) of 1.5% of the chia mucilage instead of 15% of chia seed or 1.2% of basil mucilage instead of 6% of basil seed is required to form a hydrogel. The MGR values of the mucilages were about 10% and 20% of the MGR values of the chia and basil whole seeds respectively.

**Figure 3:**
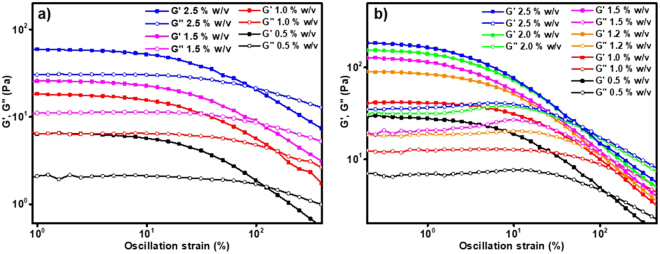
Rheology data on hydrogels of different mucilage-to-water ratios: (**a**) Chia mucilage gels, 0.5% w/v to 2.5% w/v; (**b**) Basil mucilage gel, 0.5% w/v to 2.5% w/v.

Finally, the rheological data revealed the mechanical strengths of the chia and basil gels with different mucilage-to-water ratios. As shown in Fig. 3, both chia and basil exhibited good mechanical strength in the gel state. They both showed increasing storage modulus with increasing mucilage-to-water ratio, 0.5% to 2.5% w/v. The chia gels showed storage moduli ranging from 6.69 Pa for the 0.5% gel to 59.52 Pa for the 2.5% gel. In comparison, the basil gels showed higher storage moduli ranging from 30.80 Pa for the 0.5% gel to 188.29 Pa for the 2.5% gel. The higher storage modulus seen for basil correlates with a greater affinity of its fibers for water, which is partly attributed to about 28% of the repeating unit of basil is galacturonic acid while only about 25% of the repeating unit of chia is glucuronic acid. The composition of basil mucilage is xylose, rhamnose, arabinose and galacturonic acid (15:9:7:12) whereas that of chia mucilage is xylose, glucose and glucuronic acid (2:1:1). From these ratios, we calculated basic sugar acid unit percentages. Additionally, the much enhanced storage modulus seen with basil correlates with stronger 3D network formation, which is attributed to the fact that the basic repeating unit in basil has more branches compared to that of chia (Fig. 1c). The much complex branching of the basil sugar repeating unit—with monomer, dimer and tetramer side chains—results in more efficient interaction and overlapping to form a more mechanically strong 3D network.

### Microscopic studies results

#### Optical microscopy

**Figure 4:**
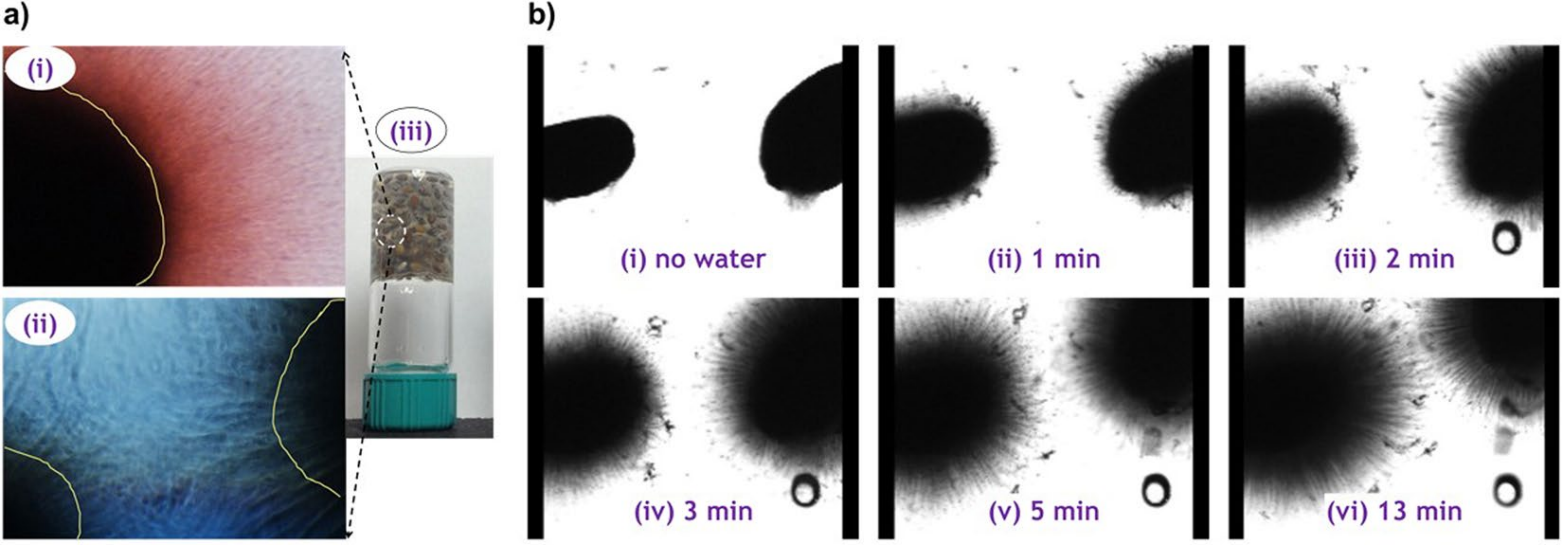
(**a**) Optical micrographs of chia seed fibers dyed with Congo Red (seen as bundle of fibers) and Methylene Blue (seen as interconnected fibers between two seeds), (i) and (ii) respectively; photograph of an inverted chia seed gel (iii); (**b**) Optical microscope video: snapshots of live fiber growth using an optical microscope: (i), no water was added; (ii–vi), number of minutes representing the duration of time elapsed after water was added.

Optical micrographs of grown chia seed fibers, along side a photograph of chia seed gel, are shown in Fig. 4a. The evenly spaced out seeds in the gel (as also seen in Fig. 1) led us to believe that there must be some sort of fibers responsible for separating the seeds apart as well as entrapping the water molecules.

The optical microscope results gave evidence of some kind of filaments extending around or between adjacent seeds. The images in Fig. 4a(i and ii) show bundles of extended fibers of part of a chia seed soaked in water-dyed with Congo red (Fig. 4a(i)) and Methylene blue (Fig. 4a(ii)). However, the difficulty to see distinct individual fibers still persisted.

Snapshots from the optical microscope live video recording of basil fiber growth are shown in Fig. 4b (also see Supporting Video S1). The figure shows two basil seeds secured in place in a well of a multi-well-plate. Figure 4b(i) shows the seeds with no water and no fibers could be seen on the seed surfaces. Upon adding water, the fibers started to grow from the surfaces of the two seeds towards one another at a remarkable speed. The snapshot of the fiber growth after 1 minute is shown in Fig. 4b(ii). With the progression of time, the fibers from one seed continued to approach those of the other seed until the fibers were almost touching/overlapping as shown by snapshots after 2, 3, 5 and 13 minutes respectively in Fig. 4b(iii to vi); see supporting information for Video S1 and Video S2 (nucleation and growth of a low-molecular weight gel 3D network) for comparison.

#### Scanning electron microscopy (SEM)

**Figure 5:**
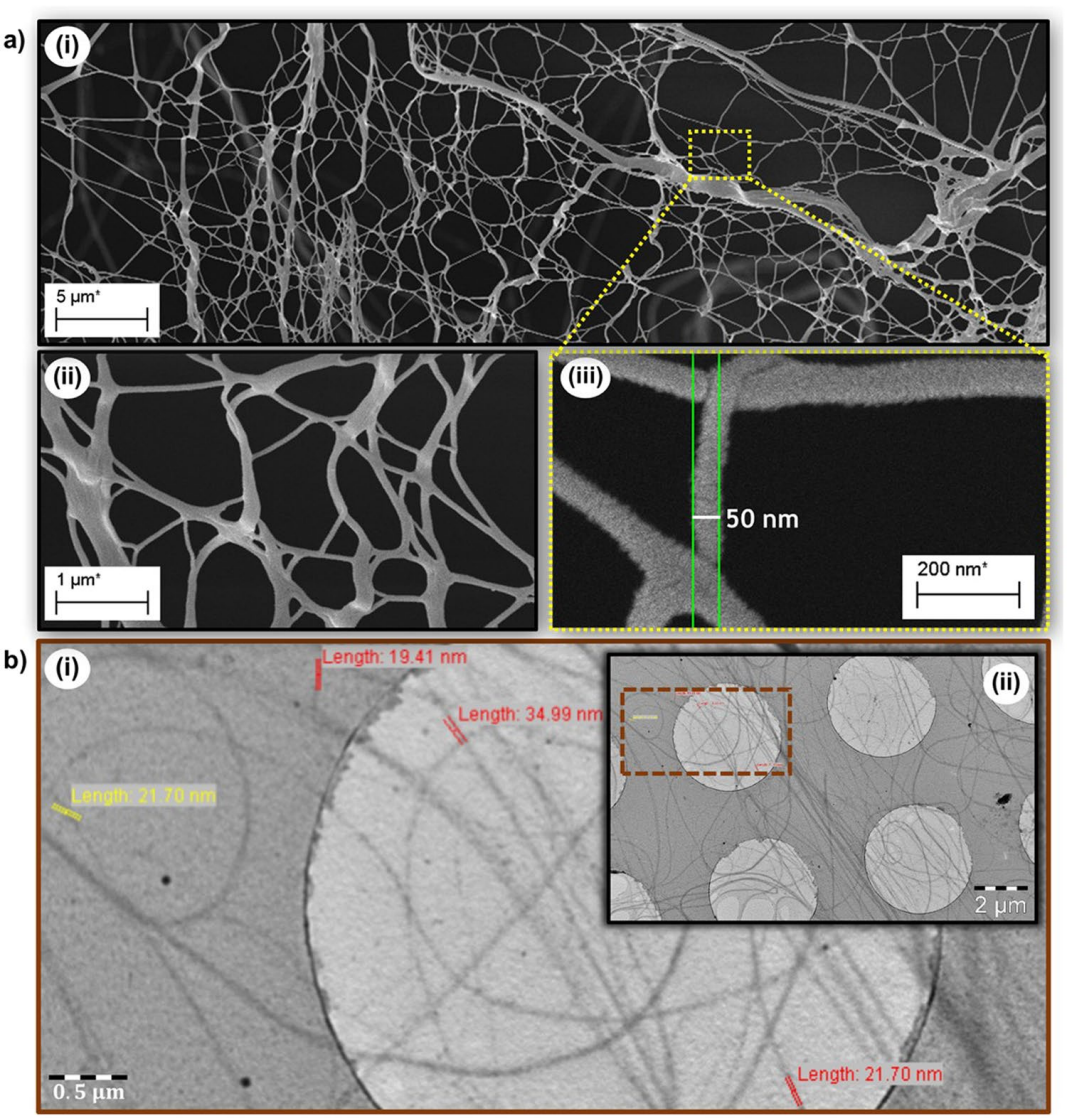
(**a**) SEM micrograph of gold-coated chia seed fibers: at (i) 4,400X magnification, (ii) 30,000X magnification, and (iii) 150,000X magnification showing fiber diameter of ~50 nm. (**b**) TEM micrograph of drop-casted aqueous solution of chia seed fibers: (i) zoomed image showing fiber diameters as low as ~20 nm, (ii) inset showing full image.

Despite optical microscopy, in conjunction with using dyes and live video recording, alluded to the nanoscopic nature of fibers responsible for gelation, it was not able to reveal distinct individual fibers or 3D network as hypothesized from the beginning; it only revealed bundles of fibers. Therefore, electron microscopy was explored for further investigation. Figure 5a shows SEM micrographs obtained using the ZEISS Supra 55 VP electron microscope. The SEM analyses resulted in an enhanced means of zooming in to deduce the nature of the fibers covering the surface of the seeds. The samples were prepared by forming chia seed hydrogel, which was then lyophilized in order to carefully void the gel of its water content while leaving the fibers in their extended state. The extremely dried, lyophilized samples were viewed under the SEM. The SEM imaging corroborated our hypothesis (Fig. 5a); we observed an extensive network of nanoscale fibers extended from proximate seeds into all directions at about 4400X magnification (Fig. 5a(i)). Furthermore, an even higher magnification (~150000X) revealed nanoscale fibres with diameters around 50 nm (Fig. 5a(iii)).

This breakthrough observation confirms our hypothesis and sheds light on the fundamental mechanism behind the gelation of these seeds at the nanoscale level. It has a major implication to the science of gelation of such seeds, since their mode of gelation had not been thoroughly scrutinized or understood^24^. The mode of gelation of unrefined materials like chia and basil seeds was only casually regarded as undergoing “coagulation” or “swelling”, although that of synthesized molecules or refined natural macromolecules has been found be via the interaction of nanoscale fibres to form a 3D network. Consequently, for the first time, our discovery of such elegant 3D structure formation by seeds like chia and basil at nanoscale level (Fig. 5a) will inevitably give more insight about how such seeds undergo gelation through the formation of 3D network of fibres at the aforementioned sublevel to induce hydrogelation. This would potentially provide grounds to study and mimic the efficiency of nature (biomimicry) to develop designed gels, 3D architectures and soft materials, as well as pave way to discovery of more gel-forming materials for targeted applications.

#### Transmission electron microscopy (TEM)

In addition to the SEM results, the transmission electron microscope (TEM) revealed a network of distinct fibres, as shown in Fig. 5b. The figure shows fibres within the vicinity of 20 nm to 30 nm in diameter. In light of these results, the nanoscopic nature of the fibres and 3D network of such mucilages that undergo hydrogelation has been further corroborated.

## Conclusion

This work has successfully shown the involvement of nanoscale 3D network and fibres in the gelation process of the mucilage of seeds like chia and basil as hypothesized, providing a link between the studied seeds’ mucilages and their known properties such as moisture retention and curbing of obesity, diabetes, and other diet-related conditions. The results were achieved via a systematic study that involved probing the influences of different parameters on gelation/partial gelation, imaging/video recording with an optical microscope, and finally imaging with scanning electron microscope (SEM) and transmission electron microscope (TEM) on samples prepared by gold coating and drop-casting on TEM grid respectively. One scientific merit of the results constitutes a more comprehensive understanding of such mucilages, especially at a time when efforts are geared towards sensitizing the public to adhere to healthy lifestyles such as consuming whole grains/fibres^18,31^. Moreover, as innovations often build upon understanding of nature, this work could be a stepping stone towards biomimetic design of next generation materials in personal care, medicine, agriculture, controlled delivery devices and cosmetics. Furthermore, the effect of ethanol on the degree of gelation/partial gelation of the mucilage could potentially be used to control the fibre extrusion in areas where the extent of fibre growth could have influence e.g. in the recovery of metals such as silver via reduction using such plant-based natural materials^32–34^.

## Materials and Methods

### Materials

Chia and basil seeds were obtained from a local store. Unless otherwise stated, all the solvents were obtained from Sigma-Aldrich and used as received. Canola oil was obtained from a local store. Congo red dye and Methylene blue dye were obtained from Sigma-Aldrich.

### Gelation and effect of various parameters

#### Gelation

Typically, gelation of the seeds was performed by adding a specific amount of the seeds (for a specific % w/v) to a vial containing the solvent. The mixture was left to settle for a few minutes and then visually verified for gelation. The usual wait time for gelation was about 15 to 20 minutes; if partial or no gel was obtained, the sample was continued to be monitored for up to 300 minutes (5 hours) just to ensure that the correct status (gel, partial gel or no gel) was assigned. Gelation was characterized by expansion of the seeds, no phase-separation, and no flow upon inverting the vial upside down. Partial gelation was characterized by the seeds becoming puffier, making the whole system significantly more viscous, instead of the seeds remaining the same or shrinking.

#### Effect of seed-to-water ratio

Different seed-to-water ratios in deionized water, as % gram of seed/mL of solvent, were systematically prepared and confirmed for gelation/partial gelation as described above. Minimum amount of seed required to gel water was determined and recorded as MGR (minimum gelation ratio).

#### Effect of solvent polarity

The effect of polarity on seed gelation was tested by using different solvent systems containing 0 to 100% ethanol in water at 10% increments. The amount of seed and solvent system used for the study was determined using the MGR value and the gelation carried out and verified as described above.

#### Effect of mode of addition of components of the mixture

How the order of adding water and ethanol components in their 50:50 mixture affected gelation was studied by either adding one (or the other) first and by adding them together. The amount of seed and solvent system used for the study was determined using the MGR value and the gelation carried out and verified as described above.

#### Effect of pH

The effect of pH on gelation was studied using pH solutions from pH 1 to pH 14. The solutions were prepared using HCl and NaOH stock solutions and the pH measured using a digital pH meter (VWR sympHony Meter, SB80PC). The amount of seed and solvent system used for the study was determined using the MGR value and the gelation carried out and verified as described above.

#### Effect of hydrophobic solvents

The effect of solvent hydrophobicity on gelation was studied by using pure hexane or canola oil. The amount of seed and solvent system used for the study was determined using the MGR value and the gelation carried out and verified as described above.

#### Mucilage extraction and gelation

A mixture of deionized water and about 15% of chia or 6% of basil seed was frozen and then freeze-dried to afford the dried, fluffy mucilage covering the surface of the seed core. The mucilage was extracted by gently rubbing the mucilage covered seeds on a metallic mesh of a strainer. Mucilage gelation was done by adding different amount of the mucilage to deionized water and then verified as described above.

#### Rheology

The mechanical strength of the chia and basil mucilage gels was studied using rheology. Different mucilage-to-water ratios ranging from 0.5 to 2.5% w/v in deionized water were used for the study. For each ratio, the linear viscosity region (LVR) was determined via amplitude strain sweep while keeping the frequency constant at 1 Hz. Next, frequency sweep was carried out while keeping the strain rate at a constant value in LVR.

### Optical microscope imaging of fibre growth

#### Optical microscope imaging of grown fibres

The samples were prepared by partially gelling the seeds in water to grow the fibres and then dyed with Congo red or Methylene blue in a 6 × 4 multiwell plate. The dyed fibre samples were viewed using the Nickon TiE optical microscope. In an attempt to see more vivid individual fibres, urea, a known hydrogen bond breaker, was added to some of the samples in addition to the dye.

#### Optical microscope live video recording of fibre growth

Chia seeds were secured with *Krazy* glue about 1 mm apart in a well of a multi-well-plate. Upon focusing the microscope, few drops of water were added to the seeds and recording immediately started using the attached camera.

### Electron microscope imaging of fibre growth

#### Scanning electron microscopy (SEM)

The fibre samples for the SEM study were prepared by gelling the seeds in water, freezing, lyophilizing, gold-coating to render the fibers conductive to the SEM electron beam. The Zeiss Supra 55 VP scanning electron microscope was used for the imaging.

#### Transmission electron microscopy (TEM)

The TEM samples were prepared by gelling the seeds in water, placing drops of the fiber solution onto TEM grids, and incubating resulting samples at about 60 °C for about 24 hrs to ensure complete drying. The ZEISS 902 transmission electron microscope was used to record the micrographs.

## Electronic supplementary material

ChiaGel 3D Network Formation

Small Molecule 3D Network Formation (comparison)
